# SMYD3: An Oncogenic Driver Targeting Epigenetic Regulation and Signaling Pathways

**DOI:** 10.3390/cancers12010142

**Published:** 2020-01-06

**Authors:** Cinzia Bottino, Alessia Peserico, Cristiano Simone, Giuseppina Caretti

**Affiliations:** 1 Department of Biosciences, Università degli Studi Milano, Via Celoria 26, 20133 Milan, Italy; 2 Faculty of Bioscience and Agro-Food and Environmental Technology, University of Teramo, 64100 Teramo, Italy; 3 Medical Genetics, National Institute of Gastroenterology, ‘S. de Bellis’ Research Hospital, Via Turi 27, Castellana Grotte, 70013 Bari, Italy; 4 Division of Medical Genetics, Department of Biomedical Sciences and Human Oncology (DIMO), University of Bari Aldo Moro, Piazza G. Cesare 11, 70124 Bari, Italy

**Keywords:** SMYD3, KMT, epigenetic inhibitors, lysine methylation

## Abstract

SMYD3 is a member of the SMYD lysine methylase family and plays an important role in the methylation of various histone and non-histone targets. Aberrant *SMYD3* expression contributes to carcinogenesis and SMYD3 upregulation was proposed as a prognostic marker in various solid cancers. Here we summarize SMYD3-mediated regulatory mechanisms, which are implicated in the pathophysiology of cancer, as drivers of distinct oncogenic pathways. We describe SMYD3-dependent mechanisms affecting cancer progression, highlighting SMYD3 interplay with proteins and RNAs involved in the regulation of cancer cell proliferation, migration and invasion. We also address the effectiveness and mechanisms of action for the currently available SMYD3 inhibitors. The findings analyzed herein demonstrate that a complex network of SMYD3-mediated cytoplasmic and nuclear interactions promote oncogenesis across different cancer types. These evidences depict SMYD3 as a modulator of the transcriptional response and of key signaling pathways, orchestrating multiple oncogenic inputs and ultimately, promoting transcriptional reprogramming and tumor transformation. Further insights into the oncogenic role of SMYD3 and its targeting of different synergistic oncogenic signals may be beneficial for effective cancer treatment.

## 1. Introduction

### Lysine Methylation and Cancer

Over the past decades, several enzymes were identified as chromatin writers of epigenetic marks. Subsequently, novel classes of erasers of these marks have been characterized, as well as of chromatin readers, which translate these chromatin marks in a functional output [1].

A number of studies and technological advances of recent years have improved our understanding of one of these histone marks, histone lysine methylation, emphasizing its fundamental role in chromatin processes, such as transcription, DNA replication and DNA repair, and revealed lysine methylation involvement in different cellular processes, such as cell fate determination and maintenance, cell signaling, genome stability and cell proliferation [2]. In particular, these insights have led to the elucidation of an extensive set of functions for lysine methylases (KMT) that can be associated with molecular events contributing to tumorigenesis, tumor maintenance and dissemination [3]. For instance, several events involving chromatin deregulation in cancer have underscored alterations in histone lysine methylation, leading to a modified transcriptional programming of the cancerous cell, thus sustaining oncogenic transformation [4].

More recently, it was appreciated that non-histone proteins are also targeted by lysine methylase and demethylase, and that the molecular function of this modification in these targets mirrors what was previously described for histones, representing a signal for association with effector proteins [5]. Overall, the current interpretation is that lysine methylation plays a similar role in histone and non-histone substrates, modulating protein–protein and protein–DNA interactions [5,6].

These studies on how KMT affect the chromatin landscape during cancer progression also recognized that these sets of chromatin factors are novel targets for therapeutic intervention, and set the ground for the development of epigenetic drugs aimed at blocking their activity and ultimately, their molecular reprogramming occurring in cancer [7].

Among the numerous lysine methylases, SMYD3 represents an interesting example because it plays a significant role in cancer progression and invasion, both methylating several non-histone proteins involved in tumorigenesis and affecting transcriptional regulation.

Here, we aim at drawing together several reports addressing SMYD3 function in cancer, as well as the diverse activities in which it is involved.

## 2. SMYD3 Structure

Five members of the SMYD family have been identified (SMYD1–5). Their distinctive features are the presence of a myeloid-Nervy-DEAF-1 (MYND) domain and a SET domain, which is split in two by the MYND domain and is followed by a cysteine-rich post-SET portion [8,9]. SMYD1–4 also share the presence of a degenerate tetratricopeptide repeat (TPR) at the carboxy terminal domain (CTD) [10].

**Figure 1:**
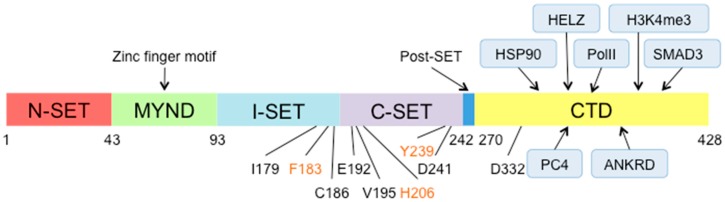
SMYD3 domains. Schematic representation of SMYD3 domains, highlighting regions of association with interactors and residues crucial for methylation activity. Residues in orange were tested both in biochemical assays and in cell culture.

As previously described, for the majority of KMTs, the SET domain of SMYD proteins catalyzes lysine methylation, but the requirement for broader specific flanking regions neighboring the SET domain is a distinctive feature of the SMYD family. In human SMYD3, the MYND domain (aa44–93), a cysteine-rich zinc finger motif, is involved in protein–protein interactions through its charged residues (Figure 1) [11]. According to other groups, this positively charged region might also serve as a DNA interaction surface, which promotes methylation activity [9]. The N-terminal region of the protein encloses a pre-SET domain (aa1–43) called N-SET domain, which acts as an inhibitory region for the enzymatic activity [12]. In fact, the deletion of the first 34 amino acids results in enhanced methylation activity [13]. The remaining part of the SET (aa94–242) follows the MYND domain and it is formed by an intermediate or linker region (I-SET) and the C-SET domain, which contains key residues for the catalytic activity (Figure 1).

In SMYD3, the TPR-like domain (aa271–428) appears to modulate the association with the MEEVD peptide within HSP90 and likely many other proteins [10,14]. The HSP90 interaction positively regulates SMYD3 in vitro methylation activity, and point mutations in residues that are crucial for HSP90 association affect SMYD3 cellular compartmentalization (Figure 1) [14].

Moreover, the CTD domain acts with the SET (aa94–242) and post-SET (aa243–270) domains, to form a deep and narrow substrate-binding pocket, showing that additional regions surrounding the SET domain are critical for substrate selection and methylase activity [9]. Several residues in the CTD region, in the SET and the post-SET domains: Y239 [15], F183 [16], C186, E192 [8], I179, V195 [17], H206 [18], D241 in SET, and D332 in CTD [12] have been identified as crucial for lysine methylation activity [9] (Figure 1).

SMYD3 crystal structure, either with substrate peptides [17], with a SAM analog [8] or with sinefungin [11] revealed its two-lobed structure surrounding the substrate binding cleft, situated in between the two lobes. The pre-SET, SET, MYND and post-SET domains constitute the N-terminal lobe, while the C-terminal domain contains the TPR region. The N-terminal lobe is crucial for cofactor binding, while the cleft formed by the two lobes creates a crevice crucial for substrate binding [19].

In addition, the CTD undergoes a hinge-like bending which reduces the clamshell opening and profiles a small substrate binding cleft [12]. According to this model, the CTD and the MYND domain may interact, further defining the narrow substrate-binding pocket [20].

Overall, structural analyses provide mechanistic insights into SMYD3 substrate preference, and its catalytic activity.

## 3. SMYD3 Levels Are Altered in Cancer

Genetic alterations of several chromatin factors endowed with oncogenic or tumor-suppressor functions affect cancer initiation and progression [21].

Experimental evidences with tumor biopsies and expression analysis of tumor datasets revealed that SMYD3 is over-expressed in various forms of cancer, and that higher SMYD3 levels correlate with a reduced overall survival and worst prognosis [22]. Recently, SMYD3 was suggested as a potential prognostic biomarker for diagnosis of prostate cancer [23], breast carcinoma (BrCA) [24] and colorectal cancer (CRC) [25].

In a comprehensive genomic and transcriptomic analysis of 51 human KMTs in a panel of breast cancer cell lines and primary breast cancer samples, SMYD3 was identified among the eight KMTs with high-level amplification and most clinical relevance [26]. Interestingly, amongst the 51 KMTs analyzed, SMYD3 showed the highest level of amplification, and the size of the SMYD3 containing 1q44 amplicon spanned approximately 1.5 Mb in breast cancer [26].

In agreement with the observation that SMYD3 is over-expressed in colorectal cancers, a study on 117 pairs of CRC and para-tumor tissues showed that the SMYD3 promoter is significantly hypomethylated in CRC when compared to the para-tumor tissues, suggesting that promoter hypomethylation may be involved in SMYD3 upregulation in certain types of cancer [25].

A homozygous [[CCGCC]_3_] variable nucleotide tandem repeat (VNTR) in the SMYD3 promoter is associated with increased risk of CRC, hepatocellular (HCC), BrCA and esophageal cancer (EC) cancer in relation to tobacco smoking [27,28,29]. In contrast, this VNTR genotype does not appear to be associated with the familial forms of BrCA and HCC cancers [30]. Importantly, the VNTR harbors a binding site for the transcription factor E2F1, which shows enhanced binding ability in the VNTR genotypes associated with different forms of cancer [28]. These data suggest that SMYD3 transcription may be regulated by E2F1 in cancer cells and that the three repeats represent a high-risk allele.

In chronic lymphocytic leukemia (CLL) cells, STAT3 is directly associated with the *SMYD3* promoter and favors SMYD3-mediated cell growth both in vivo and in vitro [31]. SMYD3 and STAT3 were found expressed at high levels in CLL. Interestingly, upregulated SMYD3 significantly promotes the CLL cells proliferation, while the inhibition of STAT3 activation dramatically reverses this effect. Furthermore, STAT3 inhibitors that prevent STAT3 phosphorylation also suppress SMYD3 expression [31].

In gastric cancer cells, Wtn3a stimulation promotes SMYD3 transcript expression, through the direct recruitment of β-catenin/TCF4 complex in Wnt3a-treated SGC7901 gastric cancer cells [32].

Insights in SMYD3 transcriptional regulation can also be gained by studies on non-cancerous models, such as in iTreg differentiation. In this system, the Notch and TGFβ pathways induce SMYD3 expression. During the early differentiation stages of iTreg, the Notch and TGFβ pathways induce *SMYD3* expression. During the early differentiation stages of iTreg, Notch signaling favors *SMYD3* expression by RBP-Jκ direct recruitment to the *SMYD3* promoter [33]. In addition, SMYD3 levels increase during 48 h of iTreg-skewing condition and TGFβ is the primary inducer of SMYD3, through the direct association of SMAD3 with the *SMYD3* promoter [34]. Nevertheless, TGFβ treatment did not induce a significant increase in *SMYD3* transcript levels in breast cancer epithelial cells [35,36], suggesting that SMAD3-dependent regulation is context specific.

Besides transcriptional regulation, SMYD3 levels are also regulated by intronic RNA, pre-mRNA and mRNA-mediated modulation.

For instance, SMYD3 intronic regions recruit the methylase EZH2, thus guiding repressive complex PRC2 association to the corresponding genomic region of *SMYD3*, resulting in increased H3K27 tri-methylation at the *SMYD3* gene and decreased levels of *SMYD3* transcripts. Thus, abnormal increase in *SMYD3* transcripts can be partially counteracted by increased recruitment of EZH2-associated repressive complexes [36].

The long noncoding RNA SPRIGHTLY (SPRIGHTLY lncRNA) was also identified as an upstream regulator of the SMYD3 pathway. SPRIGHTLY lncRNA acts as an intranuclear organizing hub for pre-mRNA molecules and its aberrant expression correlates with a variety of cancers. Lin F et al. described that this lncRNA interacts with the intronic regions of the SMYD3 pre-mRNA in the melanoma cancer cell model. Interestingly, hemizygous knockout of SPRIGHTLY by CRISPR/Cas9 in cancer cells significantly decreases SPRIGHTLY lncRNA levels, simultaneously decreasing the levels of its interacting SMYD3 pre-mRNA molecule and anchorage-independent growth rate of cells. Remarkably, the rate of in vivo tumor growth in mouse xenografts is also reduced [37].

SMYD3 levels appear to be also modulated by miRs, such as miR124 in cholangiocarcinoma cells [38] and miR346 in HCC cells [39]. miR124 directly associates with SMYD3 3’UTR and miR124 downregulation in Hepatitis C virus (HCV)-related intrahepatic cholangiocarcinoma (HCV-ICC) is linked to SMYD3 upregulation. Moreover, the migration and invasion suppressing effects of miR-124 were partially attenuated by SMYD3 over-expression [38].

miR-346 is significantly down-regulated in HCC tissues, in comparison with the non-tumor controls and is associated with the tumor size and grade. Indeed, miR-346 has a role in suppressing HCC proliferation. Bioinformatic algorithms and luciferase reporter assays proved that miR-346 directly targets the *SMYD3* 3’UTR region. Of note, down-regulation of SMYD3 neutralized the inhibitory effects of miR-346 on HCC proliferation [39].

Although all these reports (Table 1) shed some novel light on the intricate layers contributing to the regulation of SMYD3 levels, the clinical relevance of SMYD3 over-expression in cancer warrants a more comprehensive understanding of the underlying molecular mechanisms.

## 4. SMYD3-Dependent Mechanisms Affecting Cancer Progression

SMYD3 was initially described as a H3K4 methylase, involved in cancer cell proliferation [15]. However, in vitro assays with bacterially expressed SMYD3 protein revealed that SMYD3 does not display methylation activity on histone H3, neither on recombinant histone H3 nor in a nucleosomal context. Instead, in vitro methylation studies have shown that histone H4K5 is the preferred histone substrate [16,40]. However, the relevance of SMYD3-mediated H4K5 methylation is not well defined in vivo. Despite these in vitro evidences, SMYD3 knockdown has been frequently associated with a decrease in H3K4 methylation in cell culture studies. Nevertheless, SMYD3 mediated impact on H3K4 methylation may represent an indirect effect, resulting from decreased transcription of the target gene. Alternatively, SMYD3 may require other uncharacterized interacting proteins to direct its enzymatic activity on histone H3K4.

Furthermore, SMYD3 was also reported to dimethylate the histone variant H2A.Z.1 at lysine 101. The colocalization of SMYD3 and H2A.Z.1K101me2 at the promoter of cyclin A1 promotes its expression and G1-S progression in BrCA cells [41]. In agreement with these data, and with the SMYD3 role in regulating the cell cycle related gene, we have shown that SMYD3 is required for proper S phase completion in different types of tumor cells (CRC, lung, pancreatic, ovarian and prostate cancer) (Table 2) [40].

More recently, it has become clear that SMYD3 promotes oncogenesis through very diverse pathways, ascribed, on the one hand, to its enzymatic activities on histone and non-histone proteins, and on the other hand, to transcriptional related functions. In fact, SMYD3 is localized in the cytoplasm in many tumor cells [15,43], but a fraction of the protein is also found in the nucleus of several cancer cells [18,35]. These pieces of evidence suggest that SMYD3 may play different roles in distinct tumors, and that its cellular localization may be tightly regulated.

In the following sections, we will delve deeper into the diverse SMYD3-mediated functions and their role in cancer progression.

### 4.1. SMYD3 Methylates Non-Histone Proteins

SMYD3 methylation-dependent functions include its ability to methylate non-histone proteins, which are involved in the cancer cell survival and proliferation.

Specifically, SMYD3 interacts with and methylates cytoplasmic proteins, such as vascular endothelial growth factor receptor 1 (VEGFR1). The methylation of K831 potentiates angiogenesis through ligand dependent autophosphorylation and increases kinase activity [42]. In lung and pancreatic cancers, SMYD3 localization is exclusive to the cytoplasm and it potentiates RAS/ERK signaling through the methylation of the MAP3K2 kinase, by preventing its interaction with a negative regulator, namely the PP2A phosphatase complex [43]. Accordingly we found that *SMYD3* genetic knockdown correlated with reduced MEK–ERK signaling in CRC models [40]. SMYD3 over-expression has been described especially in cancer types carrying K-Ras mutations and since the k-Ras mutations occur in more than a half of human cancers, the link to SMYD3 is clinically very relevant [46,47].

Besides MAP3K2, SMYD3 was reported to methylate lysine 14 of the serine/threonine–protein kinase AKT1, a key mediator of a signaling pathway that governs various cellular processes regulating cell growth, survival, glucose metabolism, genome stability and neovascularization. Interestingly, mutation of lysine 14 diminishes the plasma membrane accumulation of AKT1. In addition, cancer cells overexpressing lysine 14-substituted AKT1 shows decreased growth rate in comparison to those overexpressing wild-type AKT1. Overall, these data imply that SMYD3-mediated methylation of AKT1 at lysine 14 is essential for AKT1 activation and suggest that the inhibition of this methylation pathway may be a promising strategy to develop anti-cancer drugs [44,45].

In breast cancer cells, SMYD3 was found to tri-methylate the HER2 protein at lysine 175, and this post-translational modification (PTM) stimulates its activation through autophosphorylation [45]. The SMYD3/HER2 link is clinically relevant because of the abnormal HER2 activation involved in development and progression of various types of cancers: namely, HER2 amplification is observed in 18–25% of BrCA and correlates with poor prognosis (Table 2) [48].

### 4.2. SMYD3 Modulates Pathways Involved in Cancer Cell Proliferation

Many studies have advocated a role for SMYD3 in the regulation of cancer cell proliferation. Interestingly, *SMYD3* knockdown by RNAi has been reported to affect cell proliferation in HCC, BrCA, cervical cancer, EC, CRC, lung adenocarcinoma and KRAS-driven pancreatic cancer cell lines [15,16,43,49,50,51,52,53,54,55,56,57]. Conversely, SMYD3 overexpression in NIH-3T3 cells has been shown to promote cell transformation by enhancing cell growth rate [15,53].

In contrast to the above findings, a recent study has shown that SMYD3 is not required for autonomous cancer cell proliferation in vitro. By means of small molecule inhibitors and CRISPR/Cas9 mutagenesis, Thomenius et al. reported the lack of proliferative effects in 240 and 313 cancer cell lines, respectively [58]. However, this study tested the in vitro effect of SMYD3 expression and/or activity impairment without taking into account the roles of tumor heterogeneity and the microenvironment.

Our previous report about SMYD3 function in tumors revealed a considerable effect on cell cycle progression with a significant increase in the S phase population, following SMYD3 genetic and pharmacological ablation. Indeed, cancer cells treated with the SMYD3 inhibitor BCI-121 accumulated in the S phase of the cell cycle, suggesting that SMYD3 might be required for proper cell cycle progression through the S/G2 boundary [40].

In agreement with our findings, Jiang Y et al. showed that SMYD3 inhibition with the small-molecule BCI-121 leads to S phase arrest and increases the ovarian cancer cell apoptosis rate. By using a gene expression array, they found several cell cycle and apoptotic modulated genes upon *SMYD3* knockdown, such as an upregulation of the cyclin-dependent kinase (CDK) inhibitors CDKN2A [p16INK4], CDKN2B [p15INK4B], CDKN3 and CDC25A, which may be responsible for the S phase arrest. In addition, the mis-regulation of the apoptotic-related genes CD40LG and BIRC3, known as apoptotic related genes, was proposed to explain the increased cell apoptosis rate following SMYD3 silencing [59]. Moreover, SMYD3 regulates the transcription of *hTERT*, a major control factor of tumor cell immortalization, through its binding to two responsive sites on the promoter of *hTERT*. SMYD3 deficient tumor cells lose occupancy of the *hTERT* promoter by the transcription factors c-MYC and Sp1, leading to repression of telomerase activity [60].

SMYD3 activity was also correlated to the expression of RIZ1, a tumor suppressor protein silenced in a variety of cancers such as liver [61], breast [62], colon [63], gastric [64] and glioma [65] and whose expression is linked to G2/M arrest and induction of apoptosis in cancer cells [66]. Interestingly, SMYD3 over-expression in HCC and esophageal squamous cells carcinoma (ESCC) was associated with *RIZ1* hypermethylation and mRNA down regulation [50,51].

In MDA-MB-231 BrCA cells, *SMYD3* knockdown induces G0/G1-phase arrest [67]. Conversely, in MGC-803 gastric cancer cells, SMYD3 down-regulation induces G2/M-phase arrest. Furthermore, it has been demonstrated that the absence of SMYD3 could halt cells in the G2/M phase via the ATM-CHK2/p53-Cdc25C pathway [57].

Taken together, these opposing data about the effect of SMYD3 on different stages of the cell cycle suggest that SMYD3 might play different roles at different stages of the cell-cycle and impact different types of carcinomas in distinct ways.

Overall, these data suggest that the combination of different SMYD3-mediated regulatory loops may affect distinct facets of proliferation in different tumor cells.

### 4.3. SMYD3 Promotes Tumor Cell Migration

Besides cell proliferation, SMYD3 has been linked to the increased migration and invasion ability of cancer cells in several tumor cell lines. Notably, SMYD3 was also identified as a signature gene in metastatic pancreatic cancer cells [68].

Cell invasion and metastatic spread are linked to a loss of epithelial factors, disruption of cell polarity and increase in mesenchymal protein expression, a process known as epithelial–mesenchymal transition (EMT) [69]. Besides their involvement in secondary tumor formation, EMT signature genes have recently been linked to cancer initiation and defined as a hallmark of cancer stem cells [70].

While a deep characterization of SMYD3 functions in different cancer stem cells is still lacking, initial findings in gastric carcinoma stem cells show that SMYD3 controls Wnt induced activation of the *ASCL2* gene, a master regulator of stem cell maintenance. SMYD3 is recruited to the chromatin regulatory region of *ASCL2* and positively regulates its transcription. Remarkably, *SMYD3* knockdown inhibits tumor sphere growth and the number of sphere-initiating cells. In vitro, SMYD3 depletion leads to decreased anchorage independent growth, and xenografts formed by SMYD3-depleted tumor cells results in smaller tumor nodules [32].

These studies represent an initial elucidation of the SMYD3 role in cancer stem cells and may pave the way to future investigations on the role of SMYD3 in the early stages of tumor formation and dissemination.

Likewise, SMYD3 over-expressing HCC cells injected in the tail vein of nude mice gave rise to a significantly higher number of metastatic lung foci when compared to control cells. In HCC, SMYD3 positive expression is significantly linked to hepatitis B virus (HBV) infection, microvascular invasion, poor tumor differentiation, high TNM stage, and a worse prognosis, suggesting that SMYD3 plays an important role in HCC progression. In cultured HCC cells, SMYD3 co-occupies the *SNAI2* promoter in collaboration with the ANKHD1 protein, promoting invasion and metastasis through the SNAIL2 pathway [71]. In addition, invasion of tumors obtained from *SMYD3* knocked-down KYSE150 ECC cells was much lower than that of control cells [72].

Among different methylases, SMYD3 is upregulated in spheroids of epithelial ovarian cancer (EOC) compared to monolayer cells, and SMYD3 depleted spheroids as well as spheroids treated with the SMYD3 inhibitor BCI-121 exhibited a decreased invasion and adhesion potential, through downregulation of integrin family members. Moreover, *SMYD3* knockdown inhibited tumor metastasis and reduced ascites volume in xenograft models [73].

SMYD3 also regulates transcription of *EZR* and *LOXL2* genes by direct recruitment to their promoter regions, thus sustaining proliferative, migration and invasion signaling in esophageal cancer cells [56]. Importantly, LOXL2 has been linked to transformation events in different types of tumors. Increased LOXL2 expression promotes gastric cancer metastasis through Src/FAK kinase, tumor progression via Snail/ E-cadherin and BrCA metastasis by activating ErbB2 via reactive oxygen species (ROS) production [74,75,76].

SMYD3 also transcriptionally regulates c-Met, a key proto-oncogene encoding a receptor tyrosine kinase [55]. c-Met is activated by the hepatocyte growth factor ligand and its overstimulation is critical in various properties of cancer cells, including proliferation and EMT induction [77]. Perturbation of SMYD3 expression affected c-Met transactivation and inhibited invasiveness of liver and BrCA cells in vitro, with mutational analysis revealing the functional importance of two SMYD3 binding sites on the c-Met promoter [55].

In prostate cancer, SMYD3 elicits its oncogenic activity by stimulating androgen receptor (*AR*) transcription. AR is the central signaling pathway in normal growth of the prostate gland and is frequently overexpressed during prostate cancer progression. Of note, genetic depletion of *SMYD3* interferes with the activation of proliferative signals and inhibits colony formation, cell migration, invasion and xenograft tumor formation. The molecular mechanism at the basis of these observations lies in the presence of two functional SMYD3-binding motifs in the *AR* promoter region [78].

In addition, SMYD3 is over-expressed in HBx-induced HCC, and it promotes invasion and metastasis through transcriptional activation of a novel oncogenic lncRNA, lncIHC [79].

Hence, SMYD3 orchestrates cancer cells proliferation and migration influencing oncogenes, tumor suppressors, cell cycle related-genes and EMT factors, thus promoting tumorigenesis in different interconnected ways.

## 5. SMYD3 Functions In Vivo

SMYD3 is ubiquitously expressed in adult tissues and its transcript is detectable during embryogenesis [80,81]. While a thorough characterization of the SMYD3 role in normal tissue is still missing, a few reports have identified a role for SMYD3 in early embryonic lineage commitment and peri-implantation development, through the modulation of lineage-specific genes [82,83]. In zebrafish, SMYD3 is required for normal cardiac and muscle development, and *SMYD3* knockdown by a morpholino approach results in embryos with pericardial edema and curved trunk [84]. The latter finding is particularly interesting, since other SMYD family members play a prominent role in myogenesis [85]. In agreement with these data, SMYD3 transcriptionally regulates myostatin and c-Met levels in mouse myotubes, thus modulating the myotubes diameter in vitro, as well as the skeletal muscle mass in vivo [86]. In addition, a recent report shows that SMYD3 modulates myoblast differentiation, through Myogenin transcriptional regulation [87]. Despite this initial characterization of SMYD3 functions in physiological conditions, SMYD3-KO (knockout) mice develop normally, and are viable and fertile [18,43], suggesting that in normal conditions, SMYD3 activities may be compensated for by other factors, for instance other SMYD family members. Although Smyd3-KO animals are healthy, the number of actively proliferating colon crypt bottom cells was reduced compared to that which was observed in control mice, and colon cells expressed significantly reduced transcript levels of *CcnA3*, *CcnD1* and *CcnE1*. These in vivo findings suggest that SMYD3 activity is critical in pathways regulating proliferation and survival abilities of normal cells. Furthermore, high expression levels of SMYD3 in tumors is linked to specific functions acquired in tumorigenesis, wherein its key role in cell proliferation is further amplified.

Conversely, studies performed with these *SMYD3* knockout mouse models have reported a crucial and specific involvement of SMYD3 in oncogenesis (Table 3). Mazur et al. showed that *SMYD3* knockdown reduces tumorigenesis induced by KRAS mutation in the pancreas and lung [43]. Likewise, Sarris et al. demonstrated that SMYD3 is required for chemically induced liver and colon carcinogenesis. In particular, the latter report showed that in a *SMYD3* total-body knockout model of diethylnitrosamine (DEN)-induced liver cancer, SMYD3 did not affect cell death and/or associated acute or chronic inflammation, but it was required for cell proliferation. In chemically induced liver tumors, SMYD3 is recruited to the promoters of cell cycle related genes (*CcnA2*, *CcnE1*, *CcnD1*, *Pcna*, and *Igfbp1*). Sarris et al. demonstrated how the expression of cell cycle related genes is marginally affected in the livers or colons of carcinogen-treated Smyd3-KO mice, as opposed to their significant upregulation in treated wild type animals, implicating a requirement for SMYD3 in carcinogenesis. ChIP- and RNA-Seq assays showed that SMYD3 directly regulates *Myc* and *Ctnnb1* oncogenes, as well as components of the IL6-Jak-Stat3 oncogenic cascade and other cell proliferation and cancer-related genes, in liver and colon carcinogenesis [18]. Notably, 66% of SMYD3 binding sites were also occupied by RNA-PolI, which is able to interact with SMYD3 in immunoprecipitation assays [15,18,86]. These findings suggest that SMYD3 recruitment to chromatin regulatory regions occurs through the association with the RNA PolII complex. In addition, SMYD3 occupancy in proximity to the transcriptional start site (TSS) matches the H3K4me3 peak distribution at TSS [18]. Moreover, in vitro data show that SMYD3 CTD can interact with a H3K4me3 peptide, suggesting that this may provide additional means of recruitment to the chromatin. SMYD3 was initially reported to be associated with the CCTCCC DNA sequence [15]. This evidence was confirmed by direct search of the sequence motifs underlying the SMYD3 binding locations [18]. Furthermore, an unbiased in vivo search revealed novel consensus sequences enriched in SMYD3 occupied regions. Overall, this genome-wide approach established that SMYD3 occupies regulatory regions of key regulators involved in proliferation, and suggested that SMYD3 recruitment to the chromatin can be mediated by its interplay with interactors, e.g., RNAPolII, H3K4me3 and putative sequence-specific DNA binding proteins. Indeed, we can imagine a scenario where SMYD3 may act as a component of multi-enzymatic complexes involved in different proliferative pathways deregulated in tumors.

This hypothesis is supported by different studies where SMYD3 has been found to interact with factors belonging to complexes that influence proliferative pathways, in cancer cell extracts. For instance, SMYD3 associates with the heat shock protein 90 (HSP90) and loss of this interaction results in impairment of the ability of SMYD3 to bind to the chromatin and to promote cell proliferation [14]. The PC4 coactivator is another component of the transcriptional machinery that interacts with SMYD3, stabilizing its chromatin occupancy and promoting the activation of cell proliferation and invasion. SMYD3 functionally cooperates with PC4, by co-localizing at target genes downstream of proliferative signals [89]. Furthermore, SMYD3 has been shown to interact with transcription factors involved in cancer, such as the estrogen receptor (ER). The ER–SMYD3 complex is recruited to the regulatory regions of ER target genes and potentiates ER-dependent gene transcription (e.g., *GREB1*, *CTSD*, *TFF1*) in the estrogen signaling pathway (Figure 1) [90,91].

In vivo, SMYD3 directly regulates key EMT inducers and regulators, in DEN-induced liver tumors and DEN-induced liver tumors in SMYD3-KO mice do not express key EMT transcripts, such as *MMP2*, *MMP14*, *Vimentin*, *Sox4*, *Zeb1*, *SNAI1* and *Fn1*, pointing to the direct role of SMYD3 in transcriptional activation [18] (Table 3).

Our data confirmed these results in an in vitro model where EMT was induced by TGFβ treatment, in BrCA cells. SMYD3 was recruited to mesenchymal gene regulatory regions (e.g., *MMP9*, *Sox9*, *SNAI1*, *Vimentin*) during TGFβ-induced EMT and SMYD3 co-occupancy with SMAD3 at these mesenchymal genes promoted transcriptional regulation. SMYD3 CTD interacts with SMAD3, and this region is crucial for mesenchymal genes regulation. Genetic knockdown or pharmacological blockade with the BC1-121 inhibitor prevents mesenchymal markers transcriptional upregulation and impairs the migration ability of TGFβ treated cells. Notably, treatment with the SMYD3 inhibitor BCI-121 reduces the invasive ability of mesenchymal breast cancer cell in vivo, in a zebrafish xenograft model [35].

Remarkably, methylation activity may be dispensable for SMYD3-mediated transcriptional regulation on certain target genes. For instance, Sarris et al. showed that a methylation-deficient SMYD3 mutant (H206G) transactivates the *Igfbp1*, *Pcna*, *Sox4*, *Myc* promoters in the same way as the methylation intact protein [18]. These findings are in agreement with our data, showing that SMYD3-mediated regulation of mesenchymal genes is also methylation independent, in TGFβ treated BrCA cells [35].

Analysis of the clinical dataset supports the results obtained in experimental models. For instance, the liver and breast human cancer sample dataset from the “The Cancer Genome Atlas” (TCGA) revealed that *SMYD3* transcript levels correlate with expression of key proliferation [18] and EMT genes [18,35]. In addition, *SMYD3* expression was positively associated, respectively, with the metalloproteinases MMP-2 and MMP-9 expression in pancreatic and gastric cancer tissues [92,93].

Remarkably, SMYD3 higher expression levels correlate with a less favorable metastasis-free survival in liver and HCC, as well as in the claudin-low BrCA subtype [18,22,35,71].

In ESCC, glioma, lung and ovarian cancers patients carrying low SMYD3-expressing tumors, overall survival [OS] is significantly longer than in those with high SMYD3-expressed cancers [22,59,88]. In ESCC, SMYD3 over-expression is also related to lymph node metastasis [56] (Table 3).

Overall, these data hint for a clinical role in SMYD3 as a prognostic factor in different forms of cancer.

## 6. Design and Testing of SMYD3 Inhibitors

The above scenario depicts how a complex network of cytoplasmic and nuclear interactions contribute to branch out SMYD3 oncogenic action across different cancer types. These data, together with the lack of obvious defects upon *SMYD3* knockout in healthy tissues (www.sanger.ac.uk/mouseportal/search?query=smyd3) and its widespread overexpression in many cancers justify the ongoing efforts in developing inhibitors blocking SMYD3 functions, as anti-cancer therapy [94].

Selective small molecules inhibitors have been designed as a tool to better characterize SMYD3 functions, and as a starting point to develop targeted therapeutic approaches. To date, few SMYD3-selective inhibitors have been described: BCI-121, EPZ031686, EPZ030456, GSK2807, EPZ02862, and tetrahydroacridine compounds as a newly designed class of irreversible inhibitors (Table 4).

BCI-121 is the first described SMYD3 inhibitor, which was identified through in silico high-throughput screening of a database. BCI-121 is associated with SMYD3 in the channel connecting the lysine and the cofactor pocket. In vitro methylation assays showed that BCI-121 blocked histone H4 methylation, blocking substrate interaction. BCI-121 treatment significantly decreased the proliferation rate of colon cancer HT29 and HCT116 cell lines, by arresting the cell cycle at S-phase.

BCI-121 binds to the histone-binding site of SMYD3 and it thus inhibits SMYD3/histone H4 interaction, in HCT116 and OVCAR-3 cancer cell lines. The SMYD3 residues interacting with BCI-121 through hydrogen bonds are S202 and Y239 in the substrate-binding pocket of SMYD3 [40]. Notably, BCI-121 administration both prevents SMYD3 enzymatic activity and SMYD3 recruitment to the chromatin of regulatory regions proliferation [35,40]. In different colon, pancreatic, ovarian and lung cancer cell lines, BCI-121 significantly reduced transcript levels of the SMYD3 target genes *c-Met*, *TERT*, *WNT10b* and *CDK2*.

GSK2807 was designed mimicking a portion of both SAH and the SMYD3 substrate MAP3K2 by blocking substrate recognition, and is a SMYD3-selective inhibitor that inhibits SMYD3 activity at IC50 value of 130 nM. Owing to the poor membrane permeability, GSK2807 cannot be used in in vivo studies. Nevertheless, it represents a powerful tool to gain insight into SMYD3 catalytic and kinetic mechanisms [96].

EPZ031686 and EPZ030456 were the first potent and selective SMYD3 inhibitors with nanomolar activity available for in vitro and cellular assays. EPZ031686 is a noncompetitive inhibitor for SAM and the MAP3K2 substrate, while EPZ030456 showed mixed type inhibition with respect to SAM, and noncompetitive inhibition with respect to MAP3K2. EPZ031686 also displayed good bioavailability in mice, making it a suitable compound for in vivo studies [95].

EPZ028862 is an isoxazole sulfonamide inhibitor, which displays potency similar to EPZ031686 in biochemical and cellular assays, and it also shows physicochemical properties that make it suitable for in vivo studies. Nevertheless, this inhibitor does not have any effect on cell proliferation in lung cancer cell lines with and without KRAS mutations, up to a concentration of 25 μM [58].

A novel class of tetrahydroacridine compounds were recently developed that block SMYD3 activity through a covalent mechanism of action. These compounds covalently modify SMYD3 via a nucleophilic aromatic substitution reaction. These molecules represent potent SMYD3 inhibitors, are chemically less reactive than classical inhibitors, and were shown to decrease cell growth in HepG2 cells. In addition, they were able to block MAP3K2 methylation in HepG2 cells [97].

Future preclinical work is warranted to show the impact of SMYD3 inhibitors treatment in tumor formation and metastasis, in mice.

## 7. Conclusions and Future Directions

**Figure 2:**
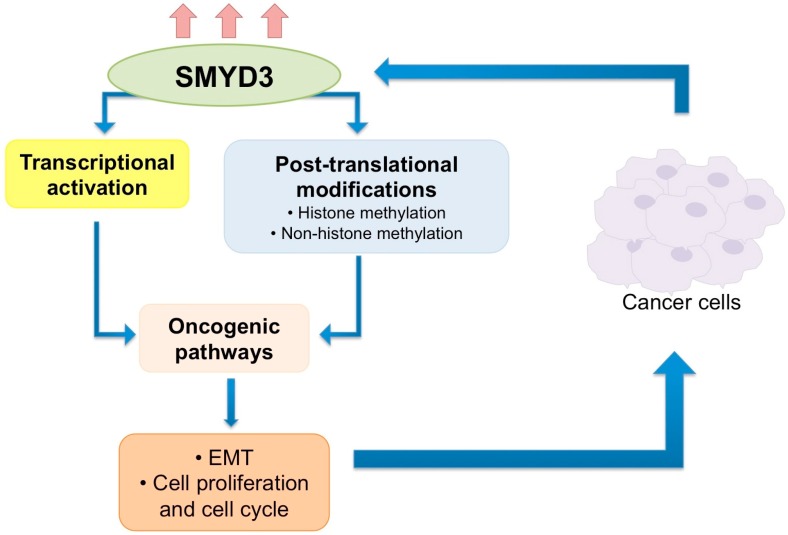
SMYD3-mediated activities that promote oncogenesis. SMYD3 is upregulated in cancer, where it promotes the activation of oncogenic pathways through two main functions, transcriptional activation of genes involved in cell proliferation and epithelial–mesenchymal transition (EMT), and through methylation of non-histone proteins, particularly involved in cell signaling, and resulting in increased cell growth and cell migration.

SMYD3 is over-expressed in several cancer types and represents a promising target to treat different malignancies. Several pathways known to be altered in tumors (e.g., beta catenin, TGFβ, STAT3) can augment SMYD3 protein levels in certain models. A more comprehensive understanding of the different mechanisms leading to SMYD3 upregulation is warranted, to define tumor-specific mechanisms resulting in SMYD3 over-expression (Figure 2).

SMYD3 appears to promote cancer in a kaleidoscopic manner, reprogramming both the transcriptional response and modulating signaling pathways, thus orchestrating different oncogenic inputs, which synergize towards tumor transformation. Likewise, SMYD3 mechanisms of action are also variegated, spanning from transcriptional regulation, which can be HMT- and non-HMT-dependent, to the PTM of proteins that is inherently HMT-dependent but can target both histone and non-histone proteins (Figure 2). Overall, these intricate regulatory networks, affecting both transcription and signaling transduction, synergize to accelerate oncogenesis, essentially through increased cell proliferation and promoting cell migration (Figure 2).

Future studies with a proteome-wide approach will lead to the identification of still unknown non-histone targets and will reveal further insights into the SMYD3-regulated signaling pathways, thus uncovering novel regulatory mechanisms. For instance, novel insights into SMYD3 post-translation modifications may also modulate its function.

These findings, when combined with additional biochemical, structural and in vivo approaches, will pave the way for the design of novel inhibitors, in the search for effective small molecules targeting SMYD3 in cancer patients.

## Figures and Tables

**Table 1:** Summary of mechanisms regulating SMYD3 levels in cancer.

Molecular Mechanism	Cancer Type	Effect on SMYD3 Levels	Ref.
1q44 ampification	Colon cancer cell lines, breast cancer	Upregulation	[26]
Promoter hypomethylation	Colon cancer	Upregulation	[25]
[(CCGCC)_3_] VNTR	Colon, hepatic, breast cancer	Upregulation	[27,28,29,30]
STAT3-mediated regulation	Chronic lymphocytic leukemia cell lines	Upregulation	[31]
Wnt-mediated regulation	Gastric cancer cell lines	Upregulation	[32]
PRC2 association to SMYD3 intronic regions	Colon cancer cell lines	Downregulation	[36]
SPRIGHTLY lncRNA	Melanoma cancer cell lines	Upregulation	[37]
miR124 downregulation	HCV-related intrahepatic cholangiocarcinoma cell lines	Upregulation	[38]
miR346 downregulation	Hepatocellular cancer cell lines	Upregulation	[39]

**Table 2:** Summary of SMYD3 methylation targets.

SMYD3 Methylation Target	Cancer Cell Line	Effect	Ref.
Histone H4-K5	HeLa and MEFs	Unknown	[16]
Histone H2A.Z.1-K101	MCF7 and TD47	Promotes transcription of proliferation related genes	[41]
VEGFR1-K831	HEK293 over-expressing SMYD3	Enhancer of VEGFR1 kinase activity	[42]
MAP3K2-260	LKR10, HEK293 over-expressing SMYD3	Methylation-dependent modulation of PP2A/MAP3K2 interaction	[43]
AKT1-K14	SW480, MDA-MB-231 HEK293 and HeLa over-expressing SMYD3	Enhancer of AKT activition	[44]
HER2-K175	none	Enhancer of HER2 activation	[45]

**Table 3:** Summary of SMYD3 roles in cancer in vivo.

Model	Cancer Type	Impact of SMYD3Increased Levels	Ref.
Mouse experimental model	pancreatic ductal and lung adenocarcinoma experimental models	Increase in MAP kinase signaling in K-Ras mutated cancers	[43]
Mouse experimental model	chemically induced liver and colon	Transcriptional up-regulation of proliferation and EMT genes	[18]
Human cancer	Breast	Reduced DFS in claudin-low patients	[35]
Human cancer	Hepatocellular carcinomas	Positive correlation with HCC development	[18]
Human cancer	Lung	Shorter progression free survival	[22]
Human cancer	Liver	Shorter overall and progression free survival	[22]
Human cancer	ESCC, ovarian, colon, glioma	Shorter overall survival	[22,25,56,59,88]

**Table 4:** Summary of SMYD3 inhibitors.

Inhibitor	Mode of Action	In Vivo Tested	Effect on Cell Models	Ref.
BCI-121	Substrate competitive	Yes	Decreased proliferation rate of colon cancer HT29 and HCT116 cell lines	[35,40]
EPZ031686	Substrate non-competitive; SAM mixed-type	Yes	-	[95]
EPZ030456	Substrate non-competitive; SAM mixed-type	No	-	[95]
GSK2807	SAM competitive	No	-	[96]
EPZ02862	Substrate non-competitive; SAM mixed-type	Yes	No effect on cell proliferation in lung cancer cell lines	[58]
Tetrahydroacridine compounds	covalent modification of SMYD3	No	Decreased proliferation of HepG2 cells	[97]

## References

[1] Lee J.S., Smith E., Shilatifard A. (2010). The language of histone crosstalk. Cell.

[2] Black J.C., Van Rechem C., Whetstine J.R. (2012). Histone lysine methylation dynamics: Establishment, regulation, and biological impact. Mol. Cell.

[3] Huang J., Berger S.L. (2008). The emerging field of dynamic lysine methylation of non-histone proteins. Curr. Opin. Genet. Dev..

[4] Dawson M.A., Kouzarides T. (2012). Cancer epigenetics: From mechanism to therapy. Cell.

[5] Cornett E.M., Ferry L., Defossez P.A., Rothbart S.B. (2019). Lysine Methylation Regulators Moonlighting outside the Epigenome. Mol. Cell.

[6] Musselman C.A., Lalonde M.E., Côté J., Kutateladze T.G. (2012). Perceiving the epigenetic landscape through histone readers. Nat. Struct. Mol. Biol..

[7] Hamamoto R., Saloura V., Nakamura Y. (2015). Critical roles of non-histone protein lysine methylation in human tumorigenesis. Nat. Rev. Cancer.

[8] Foreman K.W., Brown M., Park F., Emtage S., Harriss J., Das C., Zhu L., Crew A., Arnold L., Shaaban S. (2011). Structural and functional profiling of the human histone methyltransferase SMYD3. PLoS ONE.

[9] Xu S., Wu J., Sun B., Zhong C., Ding J. (2011). Structural and biochemical studies of human lysine methyltransferase Smyd3 reveal the important functional roles of its post-SET and TPR domains and the regulation of its activity by DNA binding. Nucleic Acids Res..

[10] Du S.J., Tan X., Zhang J. (2014). SMYD proteins: Key regulators in skeletal and cardiac muscle development and function. Anat. Rec..

[11] Sirinupong N., Brunzelle J., Doko E., Yang Z. (2011). Structural insights into the autoinhibition and posttranslational activation of histone methyltransferase SmyD3. J. Mol. Biol..

[12] Chandramouli B., Silvestri V., Scarno M., Ottini L., Chillemi G. (2016). Smyd3 open & closed lock mechanism for substrate recruitment: The hinge motion of C-terminal domain inferred from μ-second molecular dynamics simulations. Biochim. Biophys. Acta.

[13] Silva F.P., Hamamoto R., Kunizaki M., Tsuge M., Nakamura Y., Furukawa Y. (2008). Enhanced methyltransferase activity of SMYD3 by the cleavage of its N-terminal region in human cancer cells. Oncogene.

[14] Brown M.A., Foreman K., Harriss J., Das C., Zhu L., Edwards M., Shaaban S., Tucker H. (2015). C-terminal domain of SMYD3 serves as a unique HSP90-regulated motif in oncogenesis. Oncotarget.

[15] Hamamoto R., Furukawa Y., Morita M., Iimura Y., Silva F.P., Li M., Yagyu R., Nakamura Y. (2004). SMYD3 encodes a histone methyltransferase involved in the proliferation of cancer cells. Nat. Cell Biol..

[16] Van Aller G.S., Reynoird N., Barbash O., Huddleston M., Liu S., Zmoos A.F., McDevitt P., Sinnamon R., Le B., Mas G. (2012). Smyd3 regulates cancer cell phenotypes and catalyzes histone H4 lysine 5 methylation. Epigenetics.

[17] Fu W., Liu N., Qiao Q., Wang M., Min J., Zhu B., Xu R.M., Yang N. (2016). Structural Basis for Substrate Preference of SMYD3, a SET Domain-containing Protein Lysine Methyltransferase. J. Biol. Chem..

[18] Sarris M.E., Moulos P., Haroniti A., Giakountis A., Talianidis I. (2016). Smyd3 Is a Transcriptional Potentiator of Multiple Cancer-Promoting Genes and Required for Liver and Colon Cancer Development. Cancer Cell.

[19] Spellmon N., Sun X., Xue W., Holcomb J., Chakravarthy S., Shang W., Edwards B., Sirinupong N., Li C., Yang Z. (2017). New open conformation of SMYD3 implicates conformational selection and allostery. AIMS Biophys..

[20] Chandramouli B., Chillemi G. (2016). Conformational Dynamics of Lysine Methyltransferase Smyd2. Insights into the Different Substrate Crevice Characteristics of Smyd2 and Smyd3. J. Chem. Inf. Model..

[21] Shen H., Laird P.W. (2013). Interplay between the cancer genome and epigenome. Cell.

[22] Giakountis A., Moulos P., Sarris M.E., Hatzis P., Talianidis I. (2017). Smyd3-associated regulatory pathways in cancer. Semin. Cancer Biol..

[23] Vieira F.Q., Costa-Pinheiro P., Almeida-Rios D., Graça I., Monteiro-Reis S., Simões-Sousa S., Carneiro I., Sousa E.J., Godinho M.I., Baltazar F. (2015). SMYD3 contributes to a more aggressive phenotype of prostate cancer and targets Cyclin D2 through H4K20me3. Oncotarget.

[24] Song J., Liu Y., Chen Q., Yang J., Jiang Z., Zhang H., Liu Z., Jin B. (2019). Expression patterns and the prognostic value of the SMYD family members in human breast carcinoma using integrative bioinformatics analysis. Oncol. Lett..

[25] Li B., Pan R., Zhou C., Dai J., Mao Y., Chen M., Huang T., Ying X., Hu H., Zhao J. (2018). SMYD3 promoter hypomethylation is associated with the risk of colorectal cancer. Future Oncol..

[26] Liu L., Kimball S., Liu H., Holowatyj A., Yang Z.Q. (2015). Genetic alterations of histone lysine methyltransferases and their significance in breast cancer. Oncotarget.

[27] Wang X.Q., Miao X., Cai Q., Garcia-Barcelo M.M., Fan S.T. (2007). SMYD3 tandem repeats polymorphism is not associated with the occurrence and metastasis of hepatocellular carcinoma in a Chinese population. Exp. Oncol..

[28] Tsuge M., Hamamoto R., Silva F.P., Ohnishi Y., Chayama K., Kamatani N., Furukawa Y., Nakamura Y. (2005). A variable number of tandem repeats polymorphism in an E2F-1 binding element in the 5’ flanking region of SMYD3 is a risk factor for human cancers. Nat. Genet..

[29] Wang H., Liu Y., Tan W., Zhang Y., Zhao N., Jiang Y., Lin C., Hao B., Zhao D., Qian J. (2008). Association of the variable number of tandem repeats polymorphism in the promoter region of the SMYD3 gene with risk of esophageal squamous cell carcinoma in relation to tobacco smoking. Cancer Sci..

[30] Frank B., Hemminki K., Wappenschmidt B., Klaes R., Meindl A., Schmutzler R.K., Bugert P., Untch M., Bartram C.R., Burwinkel B. (2006). Variable number of tandem repeats polymorphism in the SMYD3 promoter region and the risk of familial breast cancer. Int. J. Cancer.

[31] Lin F., Wu D., Fang D., Chen Y., Zhou H., Ou C. (2019). STAT3-induced SMYD3 transcription enhances chronic lymphocytic leukemia cell growth in vitro and in vivo. Inflamm. Res..

[32] Wang T., Wu H., Liu S., Lei Z., Qin Z., Wen L., Liu K., Wang X., Guo Y., Liu Q. (2018). SMYD3 controls a Wnt-responsive epigenetic switch for ASCL2 activation and cancer stem cell maintenance. Cancer Lett..

[33] Ting H.A., de Almeida Nagata D., Rasky A.J., Malinczak C.A., Maillard I.P., Schaller M.A., Lukacs N.W. (2018). Notch ligand Delta-like 4 induces epigenetic regulation of Treg cell differentiation and function in viral infection. Mucosal. Immunol..

[34] Nagata D.E., Ting H.A., Cavassani K.A., Schaller M.A., Mukherjee S., Ptaschinski C., Kunkel S.L., Lukacs N.W. (2015). Epigenetic control of Foxp3 by SMYD3 H3K4 histone methyltransferase controls iTreg development and regulates pathogenic T-cell responses during pulmonary viral infection. Mucosal. Immunol..

[35] Fenizia C., Bottino C., Corbetta S., Fittipaldi R., Floris P., Gaudenzi G., Carra S., Cotelli F., Vitale G., Caretti G. (2018). SMYD3 promotes the epithelial-mesenchymal transition in breast cancer. Nucleic Acids Res..

[36] Guil S., Soler M., Portela A., Carrère J., Fonalleras E., Gómez A., Villanueva A., Esteller M. (2012). Intronic RNAs mediate EZH2 regulation of epigenetic targets. Nat. Struct. Mol. Biol..

[37] Lee B., Sahoo A., Marchica J., Holzhauser E., Chen X., Li J.L., Seki T., Govindarajan S.S., Markey F.B., Batish M. (2017). The long noncoding RNA. Sci. Adv..

[38] Zeng B., Li Z., Chen R., Guo N., Zhou J., Zhou Q., Lin Q., Cheng D., Liao Q., Zheng L. (2012). Epigenetic regulation of miR-124 by hepatitis C virus core protein promotes migration and invasion of intrahepatic cholangiocarcinoma cells by targeting SMYD3. FEBS Lett..

[39] Zhu W., Qian J., Ma L., Ma P., Yang F., Shu Y. (2017). MiR-346 suppresses cell proliferation through SMYD3 dependent approach in hepatocellular carcinoma. Oncotarget.

[40] Peserico A., Germani A., Sanese P., Barbosa A.J., di Virgilio V., Fittipaldi R., Fabini E., Bertucci C., Varchi G., Moyer M.P. (2015). A SMYD3 Small-Molecule Inhibitor Impairing Cancer Cell Growth. J. Cell Physiol..

[41] Tsai C.H., Chen Y.J., Yu C.J., Tzeng S.R., Wu I.C., Kuo W.H., Lin M.C., Chan N.L., Wu K.J., Teng S.C. (2016). SMYD3-Mediated H2A.Z.1 Methylation Promotes Cell Cycle and Cancer Proliferation. Cancer Res..

[42] Kunizaki M., Hamamoto R., Silva F.P., Yamaguchi K., Nagayasu T., Shibuya M., Nakamura Y., Furukawa Y. (2007). The lysine 831 of vascular endothelial growth factor receptor 1 is a novel target of methylation by SMYD3. Cancer Res..

[43] Mazur P.K., Reynoird N., Khatri P., Jansen P.W., Wilkinson A.W., Liu S., Barbash O., Van Aller G.S., Huddleston M., Dhanak D. (2014). SMYD3 links lysine methylation of MAP3K2 to Ras-driven cancer. Nature.

[44] Yoshioka Y., Suzuki T., Matsuo Y., Nakakido M., Tsurita G., Simone C., Watanabe T., Dohmae N., Nakamura Y., Hamamoto R. (2016). SMYD3-mediated lysine methylation in the PH domain is critical for activation of AKT1. Oncotarget.

[45] Yoshioka Y., Suzuki T., Matsuo Y., Tsurita G., Watanabe T., Dohmae N., Nakamura Y., Hamamoto R. (2017). Protein lysine methyltransferase SMYD3 is involved in tumorigenesis through regulation of HER2 homodimerization. Cancer Med..

[46] Forrester K., Almoguera C., Han K., Grizzle W.E., Perucho M. (1987). Detection of high incidence of K-ras oncogenes during human colon tumorigenesis. Nature.

[47] Prior I.A., Lewis P.D., Mattos C. (2012). A comprehensive survey of Ras mutations in cancer. Cancer Res..

[48] Baselga J., Swain S.M. (2009). Novel anticancer targets: Revisiting ERBB2 and discovering ERBB3. Nat. Rev. Cancer.

[49] Hamamoto R., Silva F.P., Tsuge M., Nishidate T., Katagiri T., Nakamura Y., Furukawa Y. (2006). Enhanced SMYD3 expression is essential for the growth of breast cancer cells. Cancer Sci..

[50] Chen L.B., Xu J.Y., Yang Z., Wang G.B. (2007). Silencing SMYD3 in hepatoma demethylates RIZI promoter induces apoptosis and inhibits cell proliferation and migration. World J. Gastroenterol. WJG.

[51] Dong S.W., Zhang H., Wang B.L., Sun P., Wang Y.G., Zhang P. (2014). Effect of the downregulation of SMYD3 expression by RNAi on RIZ1 expression and proliferation of esophageal squamous cell carcinoma. Oncol. Rep..

[52] Cock-Rada A.M., Medjkane S., Janski N., Yousfi N., Perichon M., Chaussepied M., Chluba J., Langsley G., Weitzman J.B. (2012). SMYD3 promotes cancer invasion by epigenetic upregulation of the metalloproteinase MMP-9. Cancer Res..

[53] Luo X.G., Ding Y., Zhou Q.F., Ye L., Wang S.Z., Xi T. (2007). SET and MYND domain-containing protein 3 decreases sensitivity to dexamethasone and stimulates cell adhesion and migration in NIH3T3 cells. J. Biosci. Bioeng..

[54] Wang S.Z., Luo X.G., Shen J., Zou J.N., Lu Y.H., Xi T. (2008). Knockdown of SMYD3 by RNA interference inhibits cervical carcinoma cell growth and invasion in vitro. BMB Rep..

[55] Zou J.N., Wang S.Z., Yang J.S., Luo X.G., Xie J.H., Xi T. (2009). Knockdown of SMYD3 by RNA interference down-regulates c-Met expression and inhibits cells migration and invasion induced by HGF. Cancer Lett..

[56] Zhu Y., Zhu M.X., Zhang X.D., Xu X.E., Wu Z.Y., Liao L.D., Li L.Y., Xie Y.M., Wu J.Y., Zou H.Y. (2016). SMYD3 stimulates EZR and LOXL2 transcription to enhance proliferation, migration, and invasion in esophageal squamous cell carcinoma. Hum. Pathol..

[57] Wang L., Wang Q.T., Liu Y.P., Dong Q.Q., Hu H.J., Miao Z., Li S., Liu Y., Zhou H., Zhang T.C. (2017). ATM Signaling Pathway Is Implicated in the SMYD3-mediated Proliferation and Migration of Gastric Cancer Cells. J. Gastric. Cancer.

[58] Thomenius M.J., Totman J., Harvey D., Mitchell L.H., Riera T.V., Cosmopoulos K., Grassian A.R., Klaus C., Foley M., Admirand E.A. (2018). Small molecule inhibitors and CRISPR/Cas9 mutagenesis demonstrate that SMYD2 and SMYD3 activity are dispensable for autonomous cancer cell proliferation. PLoS ONE.

[59] Jiang Y., Lyu T., Che X., Jia N., Li Q., Feng W. (2019). Overexpression of SMYD3 in Ovarian Cancer is Associated with Ovarian Cancer Proliferation and Apoptosis via Methylating H3K4 and H4K20. J. Cancer.

[60] Liu C., Fang X., Ge Z., Jalink M., Kyo S., Bjorkholm M., Gruber A., Sjoberg J., Xu D. (2007). The telomerase reverse transcriptase (hTERT) gene is a direct target of the histone methyltransferase SMYD3. Cancer Res..

[61] Jiang G., Liu L., Buyse I.M., Simon D., Huang S. (1999). Decreased RIZ1 expression but not RIZ2 in hepatoma and suppression of hepatoma tumorigenicity by RIZ1. Int. J. Cancer.

[62] He L., Yu J.X., Liu L., Buyse I.M., Wang M.S., Yang Q.C., Nakagawara A., Brodeur G.M., Shi Y.E., Huang S. (1998). RIZ1, but not the alternative RIZ2 product of the same gene, is underexpressed in breast cancer, and forced RIZ1 expression causes G2-M cell cycle arrest and/or apoptosis. Cancer Res..

[63] Chadwick R.B., Jiang G.L., Bennington G.A., Yuan B., Johnson C.K., Stevens M.W., Niemann T.H., Peltomaki P., Huang S., de la Chapelle A. (2000). Candidate tumor suppressor RIZ is frequently involved in colorectal carcinogenesis. Proc. Natl. Acad. Sci. USA.

[64] Piao Z., Fang W., Malkhosyan S., Kim H., Horii A., Perucho M., Huang S. (2000). Frequent frameshift mutations of RIZ in sporadic gastrointestinal and endometrial carcinomas with microsatellite instability. Cancer Res..

[65] Zhang C., Zhu Q., He H., Jiang L., Qiang Q., Hu L., Hu G., Jiang Y., Ding X., Lu Y. (2015). RIZ1: A potential tumor suppressor in glioma. BMC Cancer.

[66] Jiang G.L., Huang S. (2001). Adenovirus expressing RIZ1 in tumor suppressor gene therapy of microsatellite-unstable colorectal cancers. Cancer Res..

[67] Ren T.N., Wang J.S., He Y.M., Xu C.L., Wang S.Z., Xi T. (2011). Effects of SMYD3 over-expression on cell cycle acceleration and cell proliferation in MDA-MB-231 human breast cancer cells. Med. Oncol..

[68] Nakamura T., Fidler I.J., Coombes K.R. (2007). Gene expression profile of metastatic human pancreatic cancer cells depends on the organ microenvironment. Cancer Res..

[69] Nieto M.A., Huang R.Y., Jackson R.A., Thiery J.P. (2016). EMT: 2016. Cell..

[70] Mani S.A., Guo W., Liao M.J., Eaton E.N., Ayyanan A., Zhou A.Y., Brooks M., Reinhard F., Zhang C.C., Shipitsin M. (2008). The epithelial-mesenchymal transition generates cells with properties of stem cells. Cell.

[71] Zhou Z., Jiang H., Tu K., Yu W., Zhang J., Hu Z., Zhang H., Hao D., Huang P., Wang J. (2019). ANKHD1 is required for SMYD3 to promote tumor metastasis in hepatocellular carcinoma. J. Exp. Clin. Cancer Res..

[72] Liu X., Zheng Z., Chen C., Guo S., Liao Z., Li Y., Zhu Y., Zou H., Wu J., Xie W. (2017). Network analyses elucidate the role of SMYD3 in esophageal squamous cell carcinoma. FEBS Open Bio.

[73] Lyu T., Jiang Y., Jia N., Che X., Li Q., Yu Y., Hua K., Bast R.C., Feng W. (2019). SMYD3 promotes implant metastasis of ovarian cancer via H3K4 trimethylation of integrin promoters. Int. J. Cancer.

[74] Peng L., Ran Y.L., Hu H., Yu L., Liu Q., Zhou Z., Sun Y.M., Sun L.C., Pan J., Sun L.X. (2009). Secreted LOXL2 is a novel therapeutic target that promotes gastric cancer metastasis via the Src/FAK pathway. Carcinogenesis.

[75] Peinado H., Del Carmen Iglesias-de la Cruz M., Olmeda D., Csiszar K., Fong K.S., Vega S., Nieto M.A., Cano A., Portillo F. (2005). A molecular role for lysyl oxidase-like 2 enzyme in snail regulation and tumor progression. EMBO J..

[76] Chang J., Nicolau M.M., Cox T.R., Wetterskog D., Martens J.W., Barker H.E., Erler J.T. (2013). LOXL2 induces aberrant acinar morphogenesis via ErbB2 signaling. Breast Cancer Res..

[77] Bottaro D.P., Rubin J.S., Faletto D.L., Chan A.M., Kmiecik T.E., Vande Woude G.F., Aaronson S.A. (1991). Identification of the hepatocyte growth factor receptor as the c-met proto-oncogene product. Science.

[78] Liu C., Wang C., Wang K., Liu L., Shen Q., Yan K., Sun X., Chen J., Liu J., Ren H. (2013). SMYD3 as an oncogenic driver in prostate cancer by stimulation of androgen receptor transcription. J. Natl. Cancer Inst..

[79] Chen Z., Yu W., Zhou Q., Zhang J., Jiang H., Hao D., Wang J., Zhou Z., He C., Xiao Z. (2019). A Novel lncRNA IHS Promotes Tumor Proliferation and Metastasis in HCC by Regulating the ERK- and AKT/GSK-3β-Signaling Pathways. Mol. Ther. Nucleic Acids.

[80] Rajajeyabalachandran G., Kumar S., Murugesan T., Ekambaram S., Padmavathy R., Jegatheesan S.K., Mullangi R., Rajagopal S. (2017). Therapeutical potential of deregulated lysine methyltransferase SMYD3 as a safe target for novel anticancer agents. Expert Opin. Ther. Targets.

[81] Brown M.A., Sims R.J., Gottlieb P.D., Tucker P.W. (2006). Identification and characterization of Smyd2: A split SET/MYND domain-containing histone H3 lysine 36-specific methyltransferase that interacts with the Sin3 histone deacetylase complex. Mol. Cancer.

[82] Bai H., Li Y., Gao H., Dong Y., Han P., Yu H. (2016). Histone methyltransferase SMYD3 regulates the expression of transcriptional factors during bovine oocyte maturation and early embryonic development. Cytotechnology.

[83] Suzuki S., Nozawa Y., Tsukamoto S., Kaneko T., Imai H., Minami N. (2015). Histone methyltransferase Smyd3 regulates early embryonic lineage commitment in mice. Reproduction.

[84] Fujii T., Tsunesumi S., Yamaguchi K., Watanabe S., Furukawa Y. (2011). Smyd3 is required for the development of cardiac and skeletal muscle in zebrafish. PLoS ONE.

[85] Tracy C., Warren J.S., Szulik M., Wang L., Garcia J., Makaju A., Russell K., Miller M., Franklin S. (2018). The Smyd Family of Methyltransferases: Role in Cardiac and Skeletal Muscle Physiology and Pathology. Curr. Opin. Physiol..

[86] Proserpio V., Fittipaldi R., Ryall J.G., Sartorelli V., Caretti G. (2013). The methyltransferase SMYD3 mediates the recruitment of transcriptional cofactors at the myostatin and c-Met genes and regulates skeletal muscle atrophy. Genes Dev..

[87] Codato R., Perichon M., Divol A., Fung E., Sotiropoulos A., Bigot A., Weitzman J.B., Medjkane S. (2019). The SMYD3 methyltransferase promotes myogenesis by activating the myogenin regulatory network. Sci. Rep..

[88] Dai B., Wan W., Zhang P., Zhang Y., Pan C., Meng G., Xiao X., Wu Z., Jia W., Zhang J. (2015). SET and MYND domain-containing protein 3 is overexpressed in human glioma and contributes to tumorigenicity. Oncol. Rep..

[89] Kim J.M., Kim K., Schmidt T., Punj V., Tucker H., Rice J.C., Ulmer T.S., An W. (2015). Cooperation between SMYD3 and PC4 drives a distinct transcriptional program in cancer cells. Nucleic Acids Res..

[90] Kim H., Heo K., Kim J.H., Kim K., Choi J., An W. (2009). Requirement of histone methyltransferase SMYD3 for estrogen receptor-mediated transcription. J. Biol. Chem..

[91] Li Y., Sun L., Zhang Y., Wang D., Wang F., Liang J., Gui B., Shang Y. (2011). The histone modifications governing TFF1 transcription mediated by estrogen receptor. J. Biol. Chem..

[92] Zhu C.L., Huang Q. (2019). Overexpression of the SMYD3 Promotes Proliferation, Migration, and Invasion of Pancreatic Cancer. Dig. Dis. Sci..

[93] Liu Y., Liu H., Luo X., Deng J., Pan Y., Liang H. (2015). Overexpression of SMYD3 and matrix metalloproteinase-9 are associated with poor prognosis of patients with gastric cancer. Tumour Biol..

[94] Fabini E., Manoni E., Ferroni C., Rio A.D., Bartolini M. (2019). Small-molecule inhibitors of lysine methyltransferases SMYD2 and SMYD3: Current trends. Future Med. Chem..

[95] Mitchell L.H., Boriack-Sjodin P.A., Smith S., Thomenius M., Rioux N., Munchhof M., Mills J.E., Klaus C., Totman J., Riera T.V. (2016). Novel Oxindole Sulfonamides and Sulfamides: EPZ031686, the First Orally Bioavailable Small Molecule SMYD3 Inhibitor. ACS Med. Chem. Lett..

[96] Van Aller G.S., Graves A.P., Elkins P.A., Bonnette W.G., McDevitt P.J., Zappacosta F., Annan R.S., Dean T.W., Su D.S., Carpenter C.L. (2016). Structure-Based Design of a Novel SMYD3 Inhibitor that Bridges the SAM-and MEKK2-Binding Pockets. Structure.

[97] Huang C., Liew S.S., Lin G.R., Poulsen A., Ang M.J.Y., Chia B.C.S., Chew S.Y., Kwek Z.P., Wee J.L.K., Ong E.H. (2019). Discovery of Irreversible Inhibitors Targeting Histone Methyltransferase, SMYD3. ACS Med. Chem. Lett..

